# Patterns of Psychological Responses among the Public during the Early Phase of COVID-19: A Cross-Regional Analysis

**DOI:** 10.3390/ijerph18084143

**Published:** 2021-04-14

**Authors:** Yuen Yu Chong, Wai Tong Chien, Ho Yu Cheng, Demetris Lamnisos, Jeļena Ļubenko, Giovambattista Presti, Valeria Squatrito, Marios Constantinou, Christiana Nicolaou, Savvas Papacostas, Gökçen Aydin, Francisco J. Ruiz, Maria B. Garcia-Martin, Diana P. Obando-Posada, Miguel A. Segura-Vargas, Vasilis S. Vasiliou, Louise McHugh, Stefan Höfer, Adriana Baban, David Dias Neto, Ana Nunes da Silva, Jean-Louis Monestès, Javier Alvarez-Galvez, Marisa Paez Blarrina, Francisco Montesinos, Sonsoles Valdivia Salas, Dorottya Őri, Bartosz Kleszcz, Raimo Lappalainen, Iva Ivanović, David Gosar, Frederick Dionne, Rhonda M. Merwin, Andrew T. Gloster, Maria Karekla, Angelos P. Kassianos

**Affiliations:** 1 The Nethersole School of Nursing, Faculty of Medicine, The Chinese University of Hong Kong, Hong Kong, China; wtchien@cuhk.edu.hk (W.T.C.); hycheng@cuhk.edu.hk (H.Y.C.); 2 Department of Health Sciences, European University Cyprus, 1516 Nicosia, Cyprus; D.Lamnisos@euc.ac.cy; 3 Psychological Laboratory, Faculty of Public Health and Social Welfare, Riga Stradiņš University, LV-1007 Riga, Latvia; jelena.lubenko@rsu.lv; 4 Kore University Behavioral Lab (KUBeLab), Faculty of Human and Social Sciences, Kore University of Enna, 94100 Enna, Italy; nanni.presti@gmail.com (G.P.); valeria.squatrito@gmail.com (V.S.); 5 Department of Social Sciences, School of Humanities and Social Sciences, University of Nicosia, 2417 Nicosia, Cyprus; constantinou.m@unic.ac.cy; 6 Department of Nursing, Cyprus University of Technology, 3036 Limassol, Cyprus; c.nicolaou@cut.ac.cy; 7 Cyprus Institute of Neurology and Genetics, 1683 Nicosia, Cyprus; savvas@cing.ac.cy; 8 Department of Psychological Counseling and Guidance, Faculty of Education, Hasan Kalyoncu University, Gaziantep 27010, Turkey; gokcenaydn@gmail.com; 9 Department of Psychology, Fundación Universitaria Konrad Lorenz, Bogotà 110231, Colombia; franciscoj.ruizji@gmail.com (F.J.R.); diana.obando@unisabana.edu.co (M.A.S.-V.); 10 Faculty of Psychology, University of La Sabana, Chía 53753, Colombia; pcairns@ehealthinnovation.org (M.B.G.-M.); mariabelengarciamartin2@gmail.com (D.P.O.-P.); 11 School of Applied Psychology, University College Cork, T12 YN60 Cork, Ireland; v.vasiliou@ucc.ie; 12 School of Psychology, University College Dublin, D04 V1W8 Dublin, Ireland; louise.mchugh@ucd.ie; 13 Department of Medical Psychology, Innsbruck Medical University, 6020 Innsbruck, Austria; stefan.hoefer@i-med.ac.at; 14 Department of Psychology, Babeş-Bolyai University (UBB), 400095 Cluj-Napoca, Romania; adrianababan@psychology.ro; 15 ISPA—Instituto Universitário, APPsyCI—Applied Psychology Research Center Capabilities & Inclusion, 1149-041 Lisbon, Portugal; d.neto@campus.ul.pt; 16 Faculdade de Psicologia, Alameda da Universidade, Universidade de Lisboa, 1649-013 Lisbon, Portugal; anacatarinans@gmail.com; 17 LIP/PC2S Lab, Univ. Grenoble Alpes, 38040 Grenoble, France; jlmonestes@yahoo.fr; 18 Department of Biomedicine, Biotechnology and Public Health, University of Cadiz, 11003 Cadiz, Spain; javieralvarezgalvez@gmail.com; 19 Instituto ACT, 28036 Madrid, Spain; marisa.paez@institutoact.es; 20 Department of Psychology, European University of Madrid, 28670 Madrid, Spain; francisco.montesinos2011@gmail.com; 21 Department of Psychology and Sociology, University of Zaragoza, 50009 Zaragoza, Spain; sonsoval@unizar.es; 22 Heim Pal National Pediatric Institute, Department of Mental Health, 1089 Budapest, Hungary; oridorottya@gmail.com; 23 Bartosz Kleszcz Psychotherapy and Training, ul. Aleja Zwycięstwa 31/8, 41-200 Sosnowiec, Poland; bkleszcz@gmail.com; 24 Department of Psychology, University of Jyväskylä, FI-40014 Jyväskylä, Finland; raimo.i.lappalainen@jyu.fi; 25 Clinic for Psychiatry, Clinical Center of Montenegro, 81110 Podgorica, Montenegro; ivanovicivaa@gmail.com; 26 Department of Child, Adolescent and Developmental Neurology, University Children’s Hospital, University Medical Center, 1000 Ljubljana, Slovenia; davidgosar@yahoo.com; 27 Département de Psychologie, Université du Québec à Trois-Rivières, Trois-Rivières, QC G9A 5H7, Canada; frederickdionne.psy@gmail.com; 28 Department of Psychiatry and Behavioral Science, Duke University, Durham, NC 27708, USA; rhonda.merwin@duke.edu; 29 Division of Clinical Psychology and Intervention Science, University of Basel, 4001 Basel, Switzerland; andrew.gloster@unibas.ch; 30 Department of Psychology, University of Cyprus, 1678 Nicosia, Cyprus; mkarekla@ucy.ac.cy (M.K.); angelos.kassianos@ucl.ac.uk (A.P.K.); 31 Department of Applied Health Research, University College London, London WC1E 6BT, UK

**Keywords:** COVID-19, psychological flexibility, mental health, prosociality, survey

## Abstract

This study aimed to compare the mediation of psychological flexibility, prosociality and coping in the impacts of illness perceptions toward COVID-19 on mental health among seven regions. Convenience sampled online survey was conducted between April and June 2020 from 9130 citizens in 21 countries. Illness perceptions toward COVID-19, psychological flexibility, prosociality, coping and mental health, socio-demographics, lockdown-related variables and COVID-19 status were assessed. Results showed that psychological flexibility was the only significant mediator in the relationship between illness perceptions toward COVID-19 and mental health across all regions (all *p*s = 0.001–0.021). Seeking social support was the significant mediator across subgroups (all *p*s range = <0.001–0.005) except from the Hong Kong sample (*p* = 0.06) and the North and South American sample (*p* = 0.53). No mediation was found for problem-solving (except from the Northern European sample, *p* = 0.009). Prosociality was the significant mediator in the Hong Kong sample (*p* = 0.016) and the Eastern European sample (*p* = 0.008). These findings indicate that fostering psychological flexibility may help to mitigate the adverse mental impacts of COVID-19 across regions. Roles of seeking social support, problem-solving and prosociality vary across regions.

## 1. Introduction

The rapid spread of coronavirus 2019 (COVID-19) caused by SARS-Co-V-2 amplified by forced quarantine and national lockdowns across countries have been shown to impose profound impacts on public mental health. Several meta-analyses have highlighted that at least one-third of the populations worldwide have reported symptoms of depression, anxiety, stress, and insomnia during the early stage of the COVID-19 pandemic [1,2]. In contrast, these symptoms are more severe among people with pre-existing mental health problems [3], quarantined persons [2], COVID-19 patients [2,4], and health care professionals [5,6,7]. It is expected that the adverse mental health implications arising from the pandemic can be more prevalent and persistent than the infection itself, which deserves timely and joined global efforts for efficient and effective interventions. 

With the shift of the epicenter from Mainland China to the United States and Europe, considerable variations in managing the COVID-19 pandemic have been found across the globe. For example, in the United States, the existing public health federalism allows flexibility for the State government officials to customize pandemic responses in accordance to the unique characteristics of state populations [8]. However, this creates complications in centralizing and coordinating manpower and resources across the states for implementing timely responses to address the pandemic [9]. In contrast, countries with similar infection trends such as Germany, Austria and Switzerland, who share similar federalism systems, were able to leverage state resources to implement protective policies efficiently [10]. There is a growing public consensus in implementing and adhering to a series of COVID-19 precautionary measures such as physical distancing, event restrictions, temperature checking, and closures of schools and non-essential business [11]. In contrast, the wearing of face coverings has been less widely accepted and adopted in Europe than in Asia [12]. Apart from the aforementioned variations in COVID-19 restrictions, the pre-existing socio-economic characteristics and the capacity of health care services of each individual country may have exacerbated existing health disparities across the globe [11,13]. In view of this complexity, it is expected that people across countries and regions may have different psychological responses when facing COVID-19, implying the plausible variations of strategies in addressing mental health.

**Figure 1:**
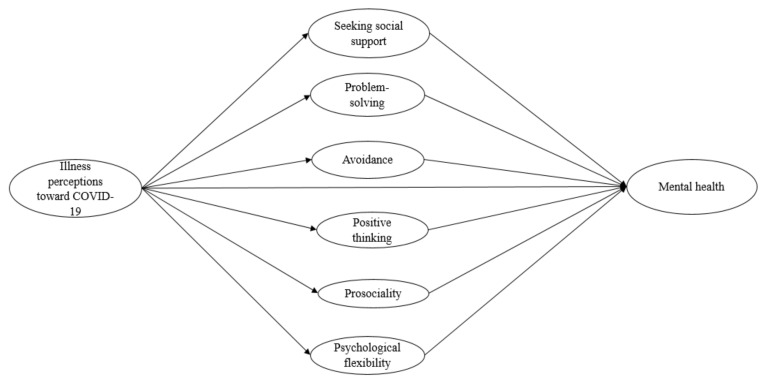
Hypothetical model of the study.

In our previous work, we followed hypotheses derived from the Leventhal’s Common Sense Model of Self-Regulation [14,15] to examine the mediating roles of coping, psychological flexibility (i.e., the capacity of being open to difficult experiences and committed toward values-driven goals [16]) and prosociality (i.e., attitudes and/or behaviors that are intended to help and benefit others [17]) in the impacts of illness perceptions toward COVID-19 on mental health [18] (see Figure 1). Using cross-sectional survey data of 514 Hong Kong adults, we found that other than those known coping factors (i.e., seeking social support, problem-solving, avoidance and positive thinking), psychological flexibility and prosociality were the two higher-order response styles that significantly mediated the impacts [18]. The purpose of the present study was to extend this single-site study evidence by investigating whether people in multiple worldwide regions exhibited similar coping patterns as of the Hong Kong sample. More specifically, in line with the theoretical bases derived from the Common Sense Model of Self-Regulation [14,15], we aimed to examine whether psychological flexibility and prosociality remained the fundamental aspects of protecting mental health among people across various geographical regions in the midst of COVID-19. In literature, only a few multi-country or multi-regional surveys have been conducted to document the prevalence of mental health illnesses in general public during the COVID-19 outbreak [19,20,21,22]. One recent meta-analysis of 55 surveys with a total of 189,159 participants has indicated that the prevalence of depression (16.2%, 34 studies) and anxiety (13.5%, 33 studies) in studies conducted in China were similar to that of studies conducted in other Asian and European countries [5] (for depression: 16.9%, 12 studies; for anxiety: 19.0%, 18 studies). However, research that focuses on comparing the psychological responses and coping patterns of people in different countries or regions when facing the pandemic is currently lacking. 

## 2. Materials and Methods

### 2.1. Study Design and Participants 

The study was an online, multi-language cross-sectional survey, COVID-19 IMPACT (see https://ucy.ac.cy/acthealthy/en/covid-19-impact-survey; accessed on 10 April 2020); its methodology of participant recruitment and data collection has been reported in our previous publications [18,23,24]. In brief, a total of 9565 individuals aged 18 years or above in 78 countries worldwide were conveniently recruited during the early phase of the COVID-19 pandemic (April to June 2020) through local press (e.g., newspapers, newsletters and radio stations), social media platforms, professional groups’ email lists and networks, as well as the participating universities’ mass emailing. These participants should be able to read at least one of the following languages (i.e., English, Greek, German, French, Spanish, Turkish, Dutch, Latvian, Italian, Portuguese, Finnish, Slovenian, Polish, Romanian, Chinese, Hungarian, Montenegrin, & Persia), and access to internet services for completing the online survey. Participants who self-selected and enrolled in the study were invited to provide informed consent and completed a 20-min online survey via a secured Google platform. 

### 2.2. Measures

The participants completed a battery of measures using the language of their choice:

1. the Mental Health Continuum Short Form for Adults (MHC-SF) assessing one’s mental health focusing on emotional, social and psychological well-being (14 items, 6-point Likert scale) [25,26];
2. the Brief Illness Perception Questionnaire (IPQ) items assessing the perceived consequences (“How much does COVID-19 affects your life?”), timeline (“How long do you think COVID-19 will continue?”), concern (“How much does COVID-19 worry you?”) and emotional responses toward COVID-19 (“How much does the pandemic COVID-19 affect you emotionally (e.g., makes you sad, angry, scared, worried”)? (4 items, 10-point Likert scale) [27];
3. the measures assessing the perceived susceptibility (3 items, 6-point Likert scale) and severity of COVID-19 (3 items, 6-point Likert scale) in line with the principles of the Health Belief Model [28];
4. the Brief Coping Orientation to Problems Experienced (Brief COPE) inventory composing of 28 items assessing a total of 14 coping strategies, which could be consolidated into four coping dimensions: seeking social support (venting, use of emotional support, use of instrumental support, religious belief); problem-solving (active coping, planning); avoidance (behavioral disengagement, self-distraction, substance use, denial, self-blaming) and positive thinking (humor, positive reframing, acceptance) [29,30,31];
5. the PsyFlex assessing all the six processes of psychological flexibility, including contacting the present moment, defusion, acceptance, self-as-context, values and committed action, of an individual (6 items, 5-point Likert scale) [32,33];
6. the Prosocialness Scale evaluating the level of prosocial behaviors, including sharing, helping, taking care of, and feeling empathic with others, which were carried out by the participant during the COVID-19 pandemic (6 items, 5-point Likert scale) [34].

The details of scoring instructions and psychometric properties of the aforementioned measures have been reported in our previous publications [18,23,24]. In summary, these measures showed satisfactory internal consistencies across participating regions (Cronbach’s alphas = 0.76–0.85) and adequate construct validity to their corresponding validation measures (*r*s = 0.68–0.82) [25,29,33,34]. In addition, the participants responded to questions about their sociodemographic characteristics, including their age, gender, country of residence, marital status, educational attainment, employment status, working as health care professionals (yes/no), and living status (living alone/others). Other COVID-19 related measures, such as the impact of lockdown on daily activities and financial situations, as well as the COVID-19 infection status of the participants (and their family members) were also assessed. The stringency of the COVID-19 precautionary measures per each participating country was tracked daily by the COVID-19 Government Response Tracker (OxCGRT). The OxCGRT was developed by the research team in the University of Oxford, which systematically summarized how the government responded in the following aspects: containment and closure such as restrictions in movement and closure of public areas (8 indicators), economic response (4 indicators) and health system polices (5 indicators) [35]. The Government Stringency Index score would then be calculated and rescaled in a range of 0 to 100, with the higher score indicating more stringent measures [35].

### 2.3. Statistical Analysis

We followed the recommendations given by the Population Division of the Department of Economic and Social Affairs of the United Nation to classify the participating countries into the following seven geographical regions: Eastern Asia, Western Asia, Northern and Southern America, Northern Europe, Western Europe, Southern Europe, and Eastern Europe [36]. Descriptive analyses and analysis of variance (ANOVA) tests were conducted to examine any significant differences on main study variables across the aforementioned regions (i.e., subgroups). The main analysis of this study consisted of testing a multiple-group structural equation model (SEM) using the SPSS AMOS version 23.0 (IBM Corp., Chicago, IL, USA) in which this model was hypothesized to illustrate the plausible mediating roles of four latent coping factors derived from the Brief COPE measure (i.e., seeking social support, problem-solving, avoidance, positive thinking), as well as the other two latent factors (i.e., prosociality and psychological flexibility) in the relationship between illness perceptions toward COVID-19 and mental health [18]. We firstly established the measurement models of all latent variables and then tested the hypothetical multiple mediation model for all the subgroups. The mediation effects were analyzed for the all the subgroups using bootstrapping method (5000 replications) with 95% bias-corrected confidence intervals. In addition, the chi-square difference test was employed to determine if there is any cross-group invariance when comparing two nested model, the unconstrained model in which no constraints were specified, and the constrained model wherein the parameters were constrained equal across the subgroups. The aforementioned SEM analyses were estimated by the maximum likelihood method, with the model fit indices (Comparative Fit Index (CFI) ≥0.90; Tucker–Lewis Index (TLI) ≥0.90; standardized root means square residual (SRMR) ≤0.10; and root mean square error approximation (RMSEA) ≤0.08) indicating an acceptable model fit [18,24]. The SEM was adjusted for the following sociodemographic variables, including age, gender, educational level, employment status and working as health care professionals (yes/no). The Government Stringency Index scores of the participating countries generated by the OxCGRT Indicators across the survey period were included as another covariate in our analysis. All statistical tests were two-sided and a *p*-value < 0.05 was considered statistically significant. 

## 3. Results

### 3.1. Descriptive Statistics

Owing to the fact that the survey was conducted online, and invitations were via social media and connections, the response rates of all study regions were unavailable. Of the 9867 respondents who accessed the survey website, 9565 provided completed data (96.9% completion rate; 88.1–100% per region). A total of 435 out of 9565 participants (4.5%) were excluded from the analysis as their corresponding countries received less than 100 completed survey responses. Table 1 summarizes the characteristics of the remaining 9130 participants in 21 countries across seven regions. The participants were mainly females (77.6%, range = 70.5–84.9% per region), middle-aged (55%, range = 43.0–64.1% per region) and employed on a full-time basis (53.7%, range = 42.2% to 63.1% per region). Less than one-fifth of the participants were health care professionals, except for those from Western Europe which composed of over 30%. More than two-thirds of the participants per region attained at least tertiary level of education. When social distancing and isolation measures began between April and June 2020 during the survey period, 47.1% of the participants stayed at home, but only 20.6% of those from Western Europe adhered to the measures. Around one-third of the participants (*n* = 3053) reported that their financial situations have got worse. A total of 133 participants (1.5%), 68 participants’ partners (0.8%) and 519 participants’ significant others (5.7%) were infected by COVID-19, respectively. Of note, the mean score of the OxCGRT Indicator in Eastern Asia (i.e., Hong Kong, mean = 59.34, SD = 8.71) was lower than that of other regions (mean range = 67.63–76.83, SD range = 7.83–14.42), indicating the implementation of COVID-19 precautionary measures by the Hong Kong government were relatively less stringent during the survey period.

Table 2 presents the illness perceptions toward COVID-19, coping, prosociality, psychological flexibility and mental health of the participants across regions. When compared with other geographical regions, analysis of variance followed by post-hoc comparisons indicated that the participants in the Eastern Asia region (i.e., Hong Kong people) reported the lowest scores in mental health (mean = 34.23, SD = 12.54, mean difference [MD] range = −4.12 to −8.81, all *p*s < 0.001), psychological flexibility (mean = 19.43, SD = 4.02, MD range = −1.7 to −3.4, all *p*s < 0.001) and prosociality (mean = 20.72, SD = 3.93, MD range = −2.12 to −3.73, all *p*s < 0.001). In addition, Hong Kong people had stronger perceptions regarding the severity of COVID-19 when compared to those in other regions (mean = 14.55, SD = 3.02, MD range = 0.99–3.43, all *p*s < 0.001). Hong Kong people also attained the highest scores in behavioral disengagement (mean = 3.40, SD = 1.26, MD range = 0.32 to 0.84, all *p*s < 0.001) and self-blaming (mean = 3.46, SD = 1.45, MD range = 0.24 to 1.56, all *p*s range = < 0.001–0.001), implying their tendencies in using maladaptive coping strategies, such as giving up to achieve goals and self-criticizing for things that happened, to manage their psychological difficulties. On the other hand, people in Western Asia reported the highest scores in active coping (mean = 6.11, SD = 1.45, MD range = 0.33 to 0.92, all *p*s < 0.001) and prosociality (mean = 24.38, SD = 3.94, MD range = 0.52 to 3.71, all *p*s < 0.001). The mental health scores across European regions (except those from Eastern Europe: mean = 37.18, SD = 14.09) were generally similar (mean range = 41.29 to 43.31, SD range = 12.98 to 14.09).

### 3.2. Model Testing and Multiple-Group Structural Equation Model Analysis

Similar to our previous reports [18,24], the measuring items corresponding to the latent constructs were all adequately fit to the data representing the total sample. The hypothetical model was first tested among the total sample and demonstrated an acceptable fit to our data (χ² = 17927.22, *df* = 629, CFI = 0.92, TLI = 0.88, SRMR = 0.05, RMSEA = 0.04), supporting that this model could be retained for subsequent multiple-group SEM analysis. Chi-square difference test showed that there was a significant difference in the model fit between the constrained model (i.e., constraining all the structural parameters in the model to be equal across the seven subgroups) and the unconstrained model (∆χ² = 2188.75, ∆*df* = 258, *p* < 0.001), indicating that the parameter coefficients differed significantly across the subgroups.

Table 3 shows the estimates of the direct and indirect effects regarding the interrelationships between illness perceptions toward COVID-19, coping and mental health based on the full unconstrained SEM model. The SEM model also showed an adequate fit to the data (χ² = 22386.96, *df* = 4403, CFI = 0.89, TLI = 0.85, SRMR = 0.05, RMSEA = 0.02). Psychological flexibility was the only factor that significantly mediated the relationship between illness perceptions toward COVID-19 and mental health across all subgroups (β range = −0.15 to −0.33, SE range = 0.04 to 0.12, all *p*s range = 0.001 to 0.021). Seeking social support showed its significant mediating role across subgroups (β range = 0.06—0.08, SE range = 0.01 to 0.03, all *p*s range = < 0.001 to 0.005) except for the Hong Kong sample (*p* = 0.06) and the North and South American sample (*p* = 0.53). Similarly, avoidance also demonstrated its significant mediating role across subgroups (β range = −0.05 to −0.32, SE range = 0.03 to 0.07, all *p*s range = < 0.001 to 0.042) except for the Eastern Europe sample (*p* = 0.07). No mediated effect was found for problem-solving (except for the Northern Europe sample, β = −0.04, SE = 0.01, *p* = 0.009). In the Hong Kong sample, prosociality (β = 0.05, SE = 0.01, *p* = 0.016) and psychological flexibility (β = −0.15, SE = 0.07, *p* = 0.021) were the core mediators of protecting mental health. The Eastern Europe sample also showed similar coping patterns, but it additionally demonstrated seeking social support as the mediator (β = 0.08, SE = 0.03, *p* = 0.005). For each subgroup SEM, the total variance explained by the predictors ranged from 56% to 73%.

## 4. Discussion

This study presents evidence indicating how coping patterns and mental health outcomes differed across various geographical regions during the early phase of COVID-19 pandemic. Our multiple group SEM analysis highlights the role of psychological flexibility as the only significant factor that mediated the relationship between illness perceptions toward COVID-19 and mental health across all included geographical regions. Indeed, several recent studies conducted in Italy [37], the United Kingdom [38,39], the United States [40,41,42], and Poland [43] have indicated how the facets of psychological flexibility play an important role in mitigating the impacts of the COVID-19 pandemic on mental health [37,38,39,40,41,43,44]. Of the aforementioned studies, some have further shown that the opposite processes of psychological flexibility, that is, the psychological inflexibility or experiential avoidance were positively associated with parenting stress and family discord [44], and moderated the suicidal risk in the context of COVID-19 stressors such as resource constraints and loss arising from the pandemic [42]. In literature, psychological flexibility has been regarded as a typical model of clinical psychological treatment, comprising the psychological processes related to acceptance, mindfulness and committed actions based on values [16,32,45]. Meta-analyses of clinical trials have highlighted the positive impacts of fostering psychological flexibility on mental health in clinical population groups such as diabetes [46], cancer [47], depression [48], anxiety spectrum disorders [48] and non-clinical population groups [49]. 

By conducting multi-group analyses, we found that despite differences in pandemic situations, social and health care contexts of the geographical regions, psychological flexibility remained as the only robust resilience factor against the adverse mental health impacts arising from COVID-19. Notably, in our study, we found that avoidance also showed a significant mediating role between illness perceptions toward COVID-19 and mental health, but it should not be considered as simply the inverse of psychological flexibility. As suggested by Dawson et al. [38], avoidance behaviors are natural human responses to an unknown threat, which can be adaptive in certain contexts (e.g., taking a short break from the sheer volume of COVID-19 related news that creates emotional disturbance [50]), but it could be a manifestation of psychological inflexibility if an individual fully engages in avoidance [38]. To summarize, our findings denote the importance of identifying, developing and evaluating a “trans-diagnostic” approach, that is, a psychotherapeutic intervention adopting the principles of acceptance, mindfulness and/or self-compassion to foster psychological flexibility for managing diverse mental health issues arising from the COVID-19 pandemic [51]. 

The significant mediating effect of seeking social support in the relationship between illness perceptions toward COVID-19 and mental health across most of the studied regions is consistent with recent evidence, supporting that increased social support has been found to protect individuals from developing mental health problems [52,53,54]. Social support refers to a series of support measures accessible to an individual through the social relationships with individuals, groups or the larger communities [55]. In literature, the benefits of social support on protecting individuals from developing mental health problems under COVID-19 has been illustrated [56,57]. Notably, the mediating role of seeking social support was not found in the participants from Eastern Asia region, which was those from Hong Kong. This result can be explained by the norm that Hong Kong people who most likely grew up under Eastern Asian culture, are less willing to seek explicit social support for dealing with stressful events [58]. If our sample could include participants from Mainland China so as to increase the representativeness of our samples under the Eastern Asia region, we might have been able to better examine whether social support could play a potential ‘protecting’ role in the detrimental mental health impacts of COVID-19 across both Western and Asian countries. In addition, no significant association was found between seeking social support and mental health in the Northern and Southern America sample, possibility due to the high levels of COVID-19 restrictions and lockdowns (as indicated by the highest mean score of the OxCGRT Indicator when compared to that of other regions) during the survey period, meaning people might have encountered difficulties in seeking direct social support from their communities.

The mediating role of prosociality as hypothesized in this study was only partially supported, as such relationship was only found in the Hong Kong and the Eastern Europe samples. In literature, studies have indicated that engaging in various forms of prosocial behaviors (i.e., helping for the benefits of others) would promote emotional well-being, empathy and social connectedness [59,60,61], while such positive impacts could be brought by mechanisms through influencing oxytocin release and reward circuitry system in the brain [61,62,63]. Furthermore, transcending self-interest to advance the welfare of others becomes an intrinsic motivation for adhering to public health measures against the COVID-19 spread (e.g., physical distancing measures, wearing a face mask, social isolation rules to protect others from COVID-19, more than that of protecting oneself) [64], or to get vaccinated against COVID-19 [65,66]. It appears that prosociality has not yet been studied and compared across multi-regional samples in the COVID-19 context, as well as outbreaks of other novel infectious diseases and disasters. This implies the need for further cross-country longitudinal studies to better understand the inter-relationships between prosociality and mental health, together with other known psychosocial and environmental factors of the pandemic. 

The mediating role of problem-solving was not found in all studied regions, except Northern Europe. Problem-solving is one of the adaptive coping strategies focusing on adapting practical steps to eliminate stress factors or reducing their impacts [67]. However, the evidence regarding whether problem-solving significantly correlates with mental health outcomes under the context of the COVID-19 pandemic remains mixed [30,68,69,70]. The non-significant result could be explained by the uncontrollable spread of the potentially fatal COVID-19, the pandemic context in which people are vulnerable to loneliness, and no effective treatments and vaccines were available at the time of survey implementation. Many people might be triggered by a sense of insecurity and inadequacy, which could be a potential stressor, and went beyond the use of problem-solving as a coping strategy to manage their psychological difficulties. 

This study had limitations. Since the online survey was administrated during the early phase of the COVID-19 pandemic (April to June 2020) and the majority of the participating countries were in partial or complete lockdown, we relied on convenience sampling in which participant recruitment was mainly carried out through social networks and various media platforms online. Hence, the representativeness of the sample has been heavily skewed to adults in European countries (i.e., 68% of the total sample). As Hong Kong was the only city out of other Eastern Asian countries or regions which participated in the survey, our findings may have limited generalizability to other Eastern Asian countries and other non-Western regions. Our convenience sampling method might not be able to reach those COVID-19 patients who had been hospitalized or are under treatment and we solely relied on self-reports, hence social desirability and response bias should be taken into account. In addition, when constructing and testing the mediational roles of coping, prosociality and psychological flexibility accounting for the relationship between illness perceptions toward COVID-19 and mental health, we followed the theoretical bases derived from the Common Sense Model of Self-Regulation for selecting and analyzing latent variables as predictors, mediators and outcomes, hence using cross-sectional data may mean we are unable to draw robust conclusions regarding the directionality of the aforementioned constructs. In each studied region, the variance to mental health contributed by psychological flexibility, prosociality and various significant coping factors ranged from 56% to 73%, but there could be other explanatory variables, such as other coping factors and self-regulatory resources, which had been missed in our study. Even though our model adjusted for sociodemographic variables and the OxCGRT indicators, the possibility of other contextual factors affecting one’s mental health, such as race, ethnicity, COVID-19 related morbidity and mortality outcomes, as well as social welfare systems across countries, cannot be ruled out. 

## 5. Conclusions

This large-scale cross-sectional survey examined how psychological flexibility, prosociality and coping mediated the impact of illness perceptions toward COVID-19 on mental health across seven geographical regions during the early phase of the pandemic. The findings pave important ways for the development of mental health interventions in navigating the current global health crisis. It is not surprising to see that people from different countries and regions exhibited different coping patterns, but they all shared the common ground in which fostering psychological flexibility played a key role in strengthening resilience. Perhaps, to support people across the globe in adapting to forthcoming COVID-19 or post-COVID-19 situational challenges, our primary health care efforts should shift to focus on fostering psychological flexibility, whether in addressing mental health needs as they arise within an individual, equipping groups (e.g., health care professionals) with skills that may foster resilience, or promoting psychological health in the broader population. One of the strategic goals determined by the World Health Organization (WHO) Special Initiative for Mental Health is the aim to increase quality and affordable community-based mental health care services for 100 million more people by 2023, so as to reduce health inequalities [71]. In addition, a recent report which summarized international experiences in the mental health response to COVID-19 has found that telehealth may soon become a core component in mental health services [72]. Hence, to maximize the reach of the psychotherapeutic interventions targeting psychological flexibility, various remote formats, such as social media platforms, mobile applications, or videoconferencing, should be adopted.

## Figures and Tables

**Table 1:** Characteristics of the participants per region.

Variables	All Regions ^a^ (*n* = 9130)	Eastern Asia—HK Only (*n* = 514)	Western Asia (*n* = 1657)	N. & S. America ^b^ (*n* = 753)	Northern Europe (*n* = 1956)	Western Europe (*n* = 1507)	Southern Europe (*n* = 1996)	Eastern Europe (*n* = 747)	χ^2^ (df)	*p*-Value
Gender, *n* (%)										
Male	2015	133	438	147	287	339	451	220	120.89 (12)	<0.001
	(22.1)	(25.9)	(26.4)	(19.5)	(14.7)	(22.5)	(22.6)	(29.5)		
Female	7084	380	1215	602	1656	1164	1540	527		
	(77.6)	(73.9)	(73.3)	(79.9)	(84.7)	(77.2)	(77.2)	(70.5)		
Non-binary	31	1	4	4	13	4	5	0		
	(0.3)	(0.2)	(0.2)	(0.5)	(0.7)	(0.3)	(0.3)	(0.0)		
Age, *n* (%)										
Young adults (18–30 years)	3532 (38.7)	259 (50.4)	882 (53.2)	362 (48.1)	545 (27.9)	533 (35.4)	604 (30.3)	347 (46.5)	443.93 (12)	<0.001
Middle-aged (31–59 years)	5017 (55.0)	245 (47.7)	727 (43.9)	324 (43.0)	1235 (63.1)	874 (58.0)	1233 (61.8)	379 (50.7)		
Older adults (≥60 years)	581 (6.4)	10 (1.9)	48 (2.9)	67 (8.9)	176 (9.0)	100 (6.6)	159 (8.0)	21 (2.8)		
Employment status, *n* (%)										
Full-time	4904 (53.7)	326 (63.4)	808 (48.8)	359 (47.7)	1288 (62.8)	610 (40.5)	1118 (56.0)	455 (60.9)	806.24 (18)	<0.001
Part-time	1599 (17.5)	72 (14.0)	189 (11.4)	115 (15.3)	266 (13.6)	557 (37.0)	323 (16,2)	77 (10.3)		
Unemployed	2028 (22.2)	103 (20.0)	583 (35.2)	222 (29.5)	267 (13.7)	256 (17.0)	428 (21.4)	169 (22.6)		
Others (retired/on leave)	599 (6.6)	13 (2.5)	77 (4.6)	57 (7.6)	1956 (10.0)	84 (5.6)	127 (6.4)	46 (6.2)		
Working as health care professionals ^b^, *n* (%)										
Yes	1478 (16.5)	59 (11.7)	168 (10.8)	57 (7.7)	204 (10.6)	473 (31.6)	388 (19.7)	129 (17.4)	400.24 (6)	<0.001
No	7472 (83.5)	444 (88.3)	1393 (89.2)	686 (92.3)	1728 (89.4)	1022 (68.4)	1586 (80.3)	613 (82.6)		
Educational level, *n* (%)										
Higher school or below	1135 (12.4)	44 (8.6)	351 (21.2)	71 (9.4)	235 (12.0)	98 (6.5)	164 (8.2)	172 (23.0)	797.19 (30)	<0.001
College/university students	1175 (12.9)	49 (9.5)	201 (12.1)	134 (17.8)	212 (10.8)	242 (16.1)	278 (13.9)	59 (7.9)		
Graduated from university	2655 (29.1)	239 (46.5)	564 (34.0)	222 (29.5)	524 (26.8)	235 (15.6)	622 (31.2)	249 (33.3)		
Master or postgraduate	3162 (34.6)	150 (29.2)	386 (23.3)	247 (32.8)	721 (36.9)	688 (45.7)	751 (37.6)	219 (29.3)		
Doctorate	764 (8.4)	32 (6.2)	132 (8.0)	59 (7.8)	240 (12.3)	153 (10.2)	115 (5.8)	33 (4.4)		
Others	239 (2.6)	0 (0.0)	23 (1.4)	20 (2.7)	24 (1.2)	91 (6.0)	66 (3.3)	15 (2.0)		
Marital status, *n* (%)										
Single	2823 (30.9)	233 (45.3)	705 (42.5)	279 (37.1)	417 (21.3)	390 (25.9)	584 (29.3)	215 (28.8)	441.82 (18)	<0.001
In a relationship/ engaged	2329 (25.5)	108 (21.0)	267 (16.1)	154 (20.5)	555 (28.4)	522 (34.6)	483 (24.2)	240 (32.1)		
Married	3297 (36.1)	163 (31.7)	618 (37.3)	26 (34.7)	758 (38.8)	499 (33.1)	761 (38.1)	237 (31.7)		
Others (divorced/widowed/separated)	681 (7.5)	10 (1.9)	67 (4.0)	59 (7.8)	226 (11.6)	96 (6.4)	168 (8.4)	55 (7.4)		
Having children, *n* (%)										
Yes	3730 (40.9)	121 (23.5)	587 (35.4)	287 (38.1)	997 (51.0)	652 (43.3)	835 (41.8)	251 (33.6)	189.86 (6)	<0.001
No	5400 (59.1)	393 (76.5)	1070 (64.6)	466 (61.9)	959 (49.0)	855 (56.7)	1161 (58.2)	496 (66.4)		
Living situation, *n* (%)										
Live alone	1341 (14.7)	38 (7.4)	186 (11.2)	79 (10.5)	351 (17.9)	270 (17.9)	291 (14.6)	126 (16.9)	1076.23 (24)	<0.001
Live with both parents	1904 (20.9)	231 (44.9)	644 (38.9)	181 (24.0)	145 (7.4)	134 (7.4)	426 (21.3)	143 (19.1)		
Living with one of the parents	465 (5.1)	34 (6.6)	85 (5.1)	86 (11.4)	94 (4.8)	44 (2.9)	83 (4.2)	39 (5.2)		
Live with own family	4928 (54.0)	179 (34.8)	695 (41.9)	365 (48.5)	1270 (64.9)	901 (59.8)	1128 (56.5)	390 (52.2)		
Live with friends/roommates	492 (5.4)	32 (6.2)	47 (2.8)	42 (5.6)	96 (4.9)	158 (10.5)	68 (3.4)	49 (6.6)		
Since the social isolation measures began, how frequent you needed to leave your house? *n* (%)										
No, I stayed at home	4304 (47.1)	173 (33.7)	803 (48.5)	504 (66.9)	889 (45.4)	310 (20.6)	1187 (59.5)	438 (58.6)	977.70 (18)	<0.001
Once only	695 (7.6)	49 (9.5)	199 (12.0)	49 (6.5)	144 (7.4)	69 (4.6)	128 (6.4)	57 (7.6)		
A couple of times	2186 (23.9)	150 (29.2)	409 (24.7)	130 (17.3)	450 (23.0)	563 (37.4)	386 (19.3)	98 (13.1)		
More than three times per week	1945 (21.3)	142 (27.6)	246 (14.8)	70 (9.3)	473 (24.2)	565 (37.5)	295 (14.8)	154 (20.6)		
Since the social isolation measures began, have your financial situation changed? *n* (%)										
Have got better	787 (8.6)	38 (7.4)	177 (10.7)	54 (7.2)	165 (8.4)	150 (10.0)	143 (7.2)	60 (8.0)	173.08 (12)	<0.001
Stay the same	5290 (57.9)	322 (62.6)	961 (58.0)	377 (50.1)	1254 (64.1)	903 (59.9)	1001 (50.2)	472 (63.2)		
Have got worse	3053 (33.4)	154 (30.0	519 (31.3)	322 (42.8)	537 (27.5)	454 (30.1)	852 (42.7)	215 (28.8)		
Have you been infected by COVID-19 ^c^? *n* (%)										
Yes	133 (1.5)	1 (0.2)	65 (3.9)	7 (0.9)	33 (1.7)	10 (0.7)	13 (0.7)	4 (0.5)	332.59 (12)	<0.001
No	8041 (88.1)	507 (98.6)	1513 (91.3)	671 (89.1)	1639 (83.8)	1254 (83.2)	1836 (92.0)	621 (83.1)		
I am not sure or have had symptoms but not diagnosed	956 (10.5)	6 (1.2)	79 (4.8)	75 (10.0)	284 (14.5)	243 (16.1)	147 (7.4)	122 (16.3)		
Have your partner being infected by COVID-19 ^c,d^? *n* (%)										
Yes	68 (0.8)	1 (0.2)	15 (0.9)	1 (0.1)	29 (1.5)	9 (0.6)	10 (0.5)	3 (0.4)	188.62 (12)	<0.001
No	8351 (92.4)	475 (98.5)	1590 (96.5)	704 (93.0)	1733 (89.2)	1320 (88.4)	1877 (94.5)	652 (88.3)		
I am not sure or have had symptoms but not diagnosed	622 (6.9)	6 (1.2)	42 (2.6)	45 (6.0)	181 (9.3)	165 (11.0)	100 (5.0)	83 (11.2)		
Have your significant others being infected by COVID-19 ^c,d^? *n* (%)										
Yes	519 (5.7)	3 (0.6)	65 (3.9)	27 (3.6)	98 (5.0)	146 (9.7)	150 (7.5)	30 (4.0)	262.11 (12)	<0.001
No	7856 (86.1)	506 (98.4)	1526 (92.1)	651 (86.5)	1655 (84.6)	1182 (78.4)	1717 (86.1)	619 (82.9)		
I am not sure or my significant others have had symptoms but not diagnosed	754 (8.3)	5 (1.0)	66 (4.0)	75 (10.0)	203 (10.4)	179 (11.9)	128 (6.4)	98 (13.1)		
COVID-19 Government Response Stringency Index (OxCGRT Indicators)										
Mean (SD) score across studied countries across study period	70.92 (6.71)	59.34 (8.71)	76.83 (12.14)	79.76 (7.83)	67.63 (18.62)	70.32 (14.42)	73.35 (13.61)	67.81 (12.42)		

^a^ In this study, Eastern Asia included Hong Kong (number of the participants, *n* = 514); Western Asia included Cyprus (*n* = 955) and Turkey (*n* = 702); Northern and Southern America included Colombia (*n* = 485) and the United States (*n* = 268); Northern Europe included The United Kingdom (*n* = 100), Finland (*n* = 157), Ireland (*n* = 414) and Latvia (*n* = 1285); Western Europe included Switzerland (*n* = 548), Germany (*n* = 278), Austria (*n* = 368) and France (*n* = 313); Southern Europe included Greece (*n* = 270), Spain (*n* = 296), Italy (*n* = 962), Portugal (*n* = 321) and Montenegro (*n* = 147); Eastern Europe included Poland (*n* = 135), Romania (*n* = 339) and Hungary (*n* = 273). ^b^ N. & S. America: Northern and Southern America. ^c^ Missing data ≤ 2%. ^d^ COVID-19: Coronavirus 2019.

**Table 2:** Illness perceptions toward COVID-19, coping, prosociality, psychological flexibility and mental health of the participants per region.

	All Regions ^a^ (*n* = 9130)	Eastern Asia—HK Only (*n* = 514)	Western Asia (*n* = 1657)	N. & S. America ^b^ (*n* = 753)	Northern Europe (*n* = 1956)	Western Europe (*n*= 1507)	Southern Europe (*n* = 1996)	Eastern Europe (*n* = 747)	*F* (*df*)	*p*-Value
Variables (Possible Range)	Mean (SD)	Mean (SD)	Mean (SD)	Mean (SD)	Mean (SD)	Mean (SD)	Mean (SD)	Mean (SD)		
Mental health										
Total score (0–70)	41.07 (13.87)	34.23 (12.54)	40.57 (14.21)	42.44 (14.89)	41.29 (13.97)	43.28 (12.98)	42.31 (13.13)	37.18 (14.09)	42.47 (6)	<0.001
Emotional (0–15)	10.15 (3.34)	8.73 (3.06)	9.60 (3.50)	10.57 (3.43)	10.42 (3.24)	10.94 (3.06)	10.23 (3.25)	9.46 (3.51)	47.98 (6)	<0.001
Social (0–25)	11.37 (5.99)	8.35 (5.05)	11.29 (6.23)	11.78 (6.24)	11.79 (5.99)	12.12 (5.81)	11.56 (5.87)	10.08 (5.70)	34.86 (6)	<0.001
Psychological (0–30)	19.55 (6.56)	17.15 (6.46)	19.69 (6.54)	20.09 (6.88)	19.08 (6.81)	20.23 (6.12)	20.51 (6.13)	17.65 (6.83)	35.24 (6)	<0.001
Illness perceptions toward COVID-19										
Consequence (1–10)	7.40 (2.24)	6.84 (1.88)	7.96 (2.12)	7.25 (2.17)	7.48 (2.21)	6.49 (2.36)	7.83 (2.07)	7.15 (2.37)	82.86 (6)	<0.001
Timeline (1–10)	6.57 (1.81)	7.09 (1.64)	7.06 (2.00)	6.78 (1.66)	6.03 (1.72)	6.34 (1.59)	6.86 (1.78)	6.03 (1.80)	85.57 (6)	<0.001
Concern (1–10)	6.72 (2.41)	6.63 (2.03)	7.30 (2.33)	7.50 (2.15)	6.65 (2.37)	5.42 (2.39)	7.36 (2.13)	5.80 (2.56)	160.04 (6)	<0.001
Emotional responses (1–10)	6.39 (2.51)	6.28 (2.09)	6.97 (2.51)	6.84 (2.45)	6.38 (2.53)	5.64 (2.49)	6.49 (2.41)	5.99 (2.60)	46.95 (6)	<0.001
Perceived susceptibility (3–18)	8.74 (3.56)	9.23 (2.99)	9.82 (3.62)	9.11 (3.63)	9.25 (3.48)	7.35 (3.27)	8.29 (3.42)	8.35 (3.66)	85.04 (6)	<0.001
Perceived severity (3–18)	12.42 (3.70)	14.55 (3.02)	11.90 (3.94)	13.56 (3.52)	12.83 (3.54)	11.12 (3.28)	12.86 (3.64)	11.37 (3.77)	101.43 (6)	<0.001
Seeking social support										
Venting (2–8)	4.76 (1.56)	5.23 (1.45)	4.93 (1.67)	4.41 (1.47)	4.46 (1.35)	5.06 (1.67)	4.52 (1.42)	5.18 (1.73)	57.49 (6)	<0.001
Use of emotional support (2–8)	4.72 (1.74)	4.75 (1.60)	4.66 (1.71)	4.43 (1.95)	4.83 (1.56)	4.76 (1.78)	4.56 (1.74)	5.26 (1.93)	20.66(6)	<0.001
Use of instrumental support (2–8)	4.38 (1.69)	5.28 (1.52)	4.88 (1.95)	4.00 (1.76)	4.34 (1.42)	4.15 (1.58)	4.08 (1.60)	4.44 (1.72)	73.93 (6)	<0.001
Religious belief (2–8)	3.84 (1.95)	4.06 (1.92)	4.69 (2.17)	4.19 (2.14)	3.56 (1.76)	3.27 (1.69)	3.61 (1.76)	3.90 (1.98)	96.39 (6)	<0.001
Problem-solving										
Active coping (2–8)	5.65 (1.55)	5.78 (1.44)	6.11 (1.45)	5.32 (1.60)	5.51 (1.45)	5.27 (1.59)	5.72 (1.53)	5.82 (1.68)	52.84 (6)	<0.001
Planning (2–8)	5.57 (1.54)	5.90 (1.34)	5.54 (1.48)	5.28 (1.64)	5.66 (1.44)	5.39 (1.64)	5.61 (1.56)	5.80 (1.61)	16.53 (6)	<0.001
Avoidance										
Behavioral disengagement (2–8)	2.86 (1.23)	3.40 (1.26)	2.99 (1.33)	2.73 (1.25)	3.06 (1.24)	2.62 (1.13)	2.69 (1.10)	2.73 (1.21)	48.51 (6)	<0.001
Self-distraction (2–8)	5.51 (1.61)	5.19 (1.54)	5.87 (1.47)	5.87 (1.49)	5.16 (1.55)	5.36 (1.76)	5.47 (1.59)	5.83 (1.67)	47.65 (6)	<0.001
Substance use (2–8)	2.60 (1.22)	2.69 (1.34)	2.37 (1.00)	2.66 (1.41)	2.82 (1.26)	2.64 (1.24)	2.37 (0.97)	3.00 (1.58)	49.09 (6)	<0.001
Denial (2–8)	2.93 (1.32)	2.84 (1.16)	3.83 (1.50)	2.60 (1.14)	2.81 (1.15)	2.45 (0.95)	2.83 (1.25)	2.91 (1.44)	199.51 (6)	<0.001
Self-blaming (2–8)	3.46 (1.45)	4.34 (1.49)	4.11 (1.47)	3.45 (1.70)	3.64 (1.39)	2.83 (1.17)	3.06 (1.24)	3.24 (1.31)	188.61 (6)	<0.001
Positive thinking										
Humor (2–8)	4.57 (1.73)	4.04 (1.54)	4.80 (1.74)	4.05 (1.87)	4.56 (1.60)	5.11 (1.75)	4.18 (1.54)	4.92 (1.93)	73.36 (6)	<0.001
Positive reframing (2–8)	5.78 (1.62)	5.50 (1.45)	6.15 (1.56)	5.31 (1.73)	5.68 (1.52)	6.10 (1.62)	5.53 (1.63)	5.93 (1.64)	48.8 9 (6)	<0.001
Acceptance (2–8)	6.55 (1.35)	6.17 (1.34)	6.17 (1.34)	6.51 (1.37)	6.72 (1.13)	6.85 (1.36)	6.61 (1.27)	6.90 (1.36)	87.54 (6)	<0.001
Psychological flexibility										
Total score (6–30)	21.83 (4.09)	19.43 (4.02)	21.15 (4.02)	21.41 (4.58)	22.09 (3.85)	22.89 (3.97)	22.22 (3.89)	21.58 (4.16)	62.57 (6)	<0.001
Prosociality										
Total score (6–30)	22.85 (4.19)	20.72 (3.93)	24.38 (3.94)	23.33 (4.25)	21.04 (4.29)	23.04 (3.66)	23.75 (3.76)	22.34 (4.31)	152.61 (6)	<0.001

^a^ In this study, Eastern Asia included Hong Kong; in Western Asia included Cyprus and Turkey; Northern and Southern America included Colombia and the United States; Northern Europe included The United Kingdom, Finland, Ireland and Latvia; Western Europe included Switzerland, Germany, Austria and France; Southern Europe included Greece, Spain, Italy, Portugal and Montenegro; Eastern Europe included Poland, Romania and Hungary. ^b^ N. & S. America: Northern and Southern America.

**Table 3:** Results of unconstrained multiple-group structural equation model by regions.

All Regions ^a ^ (*n*= 9130)	Eastern Asia, HK Only (*n* = 514)		Western Asia (*n* = 1657)		N. & S. America ^b ^ (*n* = 753)		Northern Europe *(n* = 1956)		Western Europe (*n* = 1507)		Southern Europe (*n* = 1996)		Eastern Europe (*n* = 747)	
	Β ^c^ (SE)	*p*-Value	β (SE)	*p*-Value	β (SE)	*p*-value	β (SE)	*p*-Value	β (SE)	*p*-Value	β (SE)	*p*-Value	β (SE)	*p*-Value
Direct effects from illness perceptions toward COVID-19 (i.e., unstandardized path coefficient)														
Seeking social support	0.12 (0.03)	<0.001	0.15 (0.02)	<0.001	0.10 (0.02)	<0.001	0.11 (0.01)	<0.001	0.20 (0.02)	<0.001	0.12 (0.01)	<0.001	0.21 (0.02)	<0.001
Problem-solving	−0.04 (0.03)	0.22	0.02 (0.02)	0.24	0.09 (0.02)	<0.001	0.04 (0.01)	<0.001	0.10 (0.02)	<0.001	0.12 (0.01)	<0.001	0.11 (0.03)	<0.001
Avoidance	0.10 (0.03)	<0.001	0.13 (0.01)	<0.001	0.17 (0.02)	<0.001	0.15 (0.01)	<0.001	0.14 (0.01)	<0.001	0.12 (0.01)	<0.001	0.15 (0.02)	<0.001
Positive thinking	−0.02 (0.01)	0.19	−0.03 (0.01)	<0.001	−0.03 (0.01)	0.01	−0.05 (0.01)	<0.001	−0.11 (0.01)	<0.001	−0.05 (0.01)	<0.001	−0.02 (0.01)	0.033
Prosociality	0.05 (0.02)	0.035	−0.01 (0.01)	0.29	−0.03 (0.01)	0.016	0.01 (0.01)	0.57	0.01 (0.01)	0.09	0.01 (0.01)	0.81	0.02 (0.01)	<0.001
PF ^c^	−0.12 (0.02)	<0.001	−0.10 (0.01)	<0.001	−0.14 (0.02)	<0.001	−0.12 (0.01)	<0.001	−0.09 (0.01)	<0.001	−0.10 (0.01)	<0.001	−0.13 (0.02)	<0.001
Direct effects on mental health (i.e., unstandardized path coefficient)														
Seeking social support	0.19 (0.16)	0.043	0.41 (0.12)	<0.001	−0.09 (0.14)	0.51	0.58 (0.10)	<0.001	0.41 (0.08)	<0.001	1.10 (0.20)	<0.001	0.38 (0.14)	0.004
Problem-solving	−0.57 (.66)	0.39	0.08 (0.11)	0.49	0.18 (0.50)	0.72	−0.84 (0.28)	0.008	−0.21 (0.11)	0.06	−0.40 (0.29)	0.17	−0.06 (0.33)	0.86
Avoidance	−0.68 (0.26)	0.010	−0.41 (0.20)	0.041	−0.74 (0.34)	0.03	−0.72 (0.25)	0.003	−10.02 (0.34)	0.003	−30.07 (0.60)	<0.001	−0.60 (0.48)	0.21
Positive thinking	2.83 (2.14)	0.19	0.57 (0.20)	0.005	0.70 (2.14)	0.76	1.99 (0.80)	0.013	0.67 (0.20)	<0.001	1.69 (0.46)	<0.001	1.75 (1.40)	0.21
Prosociality	0.48 (0.22)	0.029	.66 (0.13)	<0.001	0.36 (0.17)	0.030	0.16 (0.10)	0.12	0.30 (0.11)	0.023	0.59 (0.14)	<0.001	0.41 (0.16)	0.01
PF ^d^	1.30 (0.28)	<0.001	2.43 (0.18)	<0.001	2.35 (0.38)	<0.001	2.45 (0.26)	<0.001	2.53 (0.26)	<0.001	1.15 (0.35)	0.001	2.58 (0.29)	<0.001
IP ^e^	−0.09 (0.06)	0.13	−0.01 (0.03)	0.89	0.07 (0.10)	0.44	0.10 (0.06)	0.10	0.11 (0.04)	0.009	0.10 (0.07)	0.20	−0.04 (0.07)	0.55
Indirect effects														
IP➔SS ^f^➔MH ^g^	0.03 (0.02)	0.06	0.06 (0.02)	0.002	−0.01 (0.01)	0.53	0.07 (0.01)	<0.001	0.07 (0.02)	<0.001	0.12 (0.03)	<0.001	0.08 (0.03)	0.005
IP➔PS ^h^➔MH	0.02 (0.03)	0.47	0.01 (0.01)	0.56	0.02 (0.05)	0.72	−0.04 (0.01)	0.009	−0.02 (0.01)	0.12	−0.08 (0.03)	0.16	−0.01 (0.03)	0.72
IP➔Avoidance ➔MH	−0.07 (0.03)	0.04	−0.05 (0.03)	0.042	−0.13 (0.06)	0.035	−0.14 (0.04)	0.002	−0.12 (0.04)	0.004	−0.32 (0.07)	<0.001	−0.13 (0.07)	0.07
IP➔PT ^i^➔MH	−0.06 (0.05)	0.30	−0.02 (0.01)	0.038	−0.01 (0.06)	0.75	−0.11 (0.04)	0.01	−0.07 (0.03)	0.015	−0.11 (0.03)	0.011	−0.05 (0.03)	0.14
IP➔Prosociaity➔MH	0.05 (0.01)	0.016	−0.01 (0.01)	0.33	−0.01 (0.01)	0.08	0.00 (0.00)	0.39	0.00 (0.00)	0.39	−0.01 (0.01)	0.33	0.10 (0.03)	0.008
IP➔PF➔ MH	−0.15 (0.07)	0.021	−0.24 (0.03)	0.002	−0.33 (0.12)	0.02	−0.29 (0.05)	0.002	−0.25 (0.04)	0.001	−0.16 (0.05)	0.008	−0.25 (0.05)	0.004
Total variance (R^2^)	0.64		0.56		0.67		0.72		0.66		0.73		0.72	

^a^ In this study, Eastern Asia included Hong Kong; in Western Asia included Cyprus and Turkey; Northern and Southern America included Colombia and the United States; Northern Europe included The United Kingdom, Finland, Ireland and Latvia; Western Europe included Switzerland, Germany, Austria and France; Southern Europe included Greece, Spain, Italy, Portugal and Montenegro; Eastern Europe included Poland, Romania and Hungary. ^b^ N. & S. America: Northern and Southern America. ^c^ β: Unstandardized beta coefficient. ^d^ PF: Psychological flexibility. ^e^ IP: Illness perception toward COVID-19. ^f^ SS: Seeking social support. ^g^ MH: Mental health. ^h^ PS: Problem-solving. ^i^ PT: Positive thinking.

## Data Availability

The datasets used and/or analyzed during the current study are available from the corresponding author on reasonable request.
